# Development and psychometric testing of a pediatric chronic graft-versus-host disease symptom scale: protocol for a two-phase, mixed methods study

**DOI:** 10.3389/fpsyg.2023.1243005

**Published:** 2024-01-08

**Authors:** Sandra A. Mitchell, Rachael Hunter, Abigail Fry, Steven Z. Pavletic, Brigitte C. Widemann, Lori Wiener

**Affiliations:** ^1^Outcomes Research Branch, Healthcare Delivery Research Program, National Cancer Institute, Bethesda, MD, United States; ^2^Pediatric Oncology Branch, Center for Cancer Research, National Cancer Institute, Bethesda, MD, United States; ^3^Immune Deficiency Cellular Therapy Program, Center for Cancer Research, National Cancer Institute, Bethesda, MD, United States

**Keywords:** chronic graft-versus-host disease, pediatrics, hematopoietic stem cell transplant, symptom scale, patient-reported outcomes, symptom bother, validation

## Abstract

**Background:**

Chronic graft-versus-host disease (cGVHD) is a debilitating late complication of hematopoietic stem cell transplantation. It is often accompanied by extensive symptom burden. No validated cGVHD patient-reported outcome (PRO) measure exists to evaluate cGVHD symptom bother in children and adolescents younger than 18 years. This paper presents the study protocol for a multi-center, two-phase protocol to develop a psychometrically valid pediatric cGVHD Symptom Scale (PCSS) and a companion caregiver-proxy measure to capture the symptom burden experienced by children with cGVHD. In the first phase of the study, our aim is to evaluate the comprehension, clarity and ease of response of the PCSS through cognitive interviewing and to iteratively refine the measure to optimize content validity. In the second phase of the study, we will quantitatively examine the measurement properties of the PCSS in children and their caregiver-proxies.

**Methods and analysis:**

Eligible participants are children/adolescents ages 5–17 with cGVHD who are receiving systemic immunosuppressive treatment or have recently tapered to discontinuation. In the first phase, we are enrolling 60 child and caregiver-proxy dyads in three child age strata (5–7, 8–12, and 13–17 years old). Semi-scripted cognitive debriefing interviews are conducted to assess comprehension, clarity, and ease of response of each PCSS item with the child alone, and then jointly with the caregiver-proxy to explore discordant ratings. In phase two, an age-stratified cohort of 120 child-caregiver dyads will be enrolled to evaluate test–retest reliability, construct validity, and responsiveness. Anchors for known-groups validity include the PedsQL module and clinical variables, including cGVHD clinician-rated severity scores. In participants ages 13–17, we will also compare responses on the PCSS with those from the Lee cGVHD Symptom Scale, to gauge the youngest age at which adolescent respondents can comprehend this adult measure.

**Discussion:**

This study will yield a well-validated, counterpart measure to the Lee cGVHD Symptom Scale for use in children with cGVHD and their caregiver-proxies. This new patient-reported outcome measure can be integrated into clinical trials and care delivery for pediatric transplant survivors to improve the precision and accuracy with which their cGVHD symptom experience is captured.

**Clinical trial registration:**

www.ClinicalTrials.gov, NCT04044365.

## Introduction

Hematopoietic stem cell transplantation (HSCT) is a curative option for many children with cancer and certain genetic disorders. However, approximately 40% of stem cell transplant recipients experience chronic graft-versus-host disease (cGVHD), a late complication of treatment that causes burdensome symptoms, functional impairment and diminished quality of life (Agh et al., 2019).

Chronic GVHD can involve almost every organ system although it most commonly affects the skin, eyes, mouth, liver, intestines, lungs, and the musculoskeletal system (Haroun et al., 2023). Children living with cGVHD often face many years of a disabling and painful chronic illness compounded by the side effects of prolonged immunosuppression, particularly long-term corticosteroids, needed to treat cGVHD manifestations (Mitchell et al., 2021).

There has been a sustained effort through the NIH cGVHD Consensus Consortium to standardize the diagnosis, severity grading and criteria for defining therapeutic response for patients with cGVHD on clinical trials (Carpenter et al., 2015; Jagasia et al., 2015; Lee et al., 2015; Cuvelier et al., 2019; Kitko et al., 2021). However, since objective changes in cGVHD-related organ system manifestations may not fully reflect therapeutic response or subtle but clinically meaningful improvements, the NIH Consensus Panel has recommended evaluation of both the clinical signs of chronic GVHD, and also that the patient experience of cGVHD symptoms be captured by self-report using well-validated patient-reported outcomes (PRO; Lee et al., 2015, 2022). In addition, PROs can be used as a clinical tool to optimize symptom management, promote communication and shared decision-making with clinicians, and increase patient and family engagement in care, all ultimately leading to better clinical outcomes (Bele et al., 2020; Leahy and Steineck, 2020).

The Lee cGVHD Symptom Score (LSS) has been recognized as a core cGVHD outcome measure in the adult population (Lee et al., 2002, 2015; Merkel et al., 2016). However, the instrument has not been validated for use in children/adolescents younger than 18, and the phrasing of many of the symptom terms (such as limited joint movement and thickened skin) used in the LSS may not be well understood by younger children. There is currently no available pediatric PRO measure of cGVHD symptom bother.

To ensure the developmental appropriateness and strong measurement properties of pediatric PRO measures, the International Society for Pharmacoeconomics and Outcomes Research (ISPOR) and the International Society for Quality of Life Research (ISOQOL) recommends the use of both qualitative and quantitative methods to develop, refine, and test new PRO measures for this patient population (Matza et al., 2013; Reeve et al., 2013). This two phase study builds upon our prior work conducting concept elicitation interviews to identify the concepts and specific phrasings that children and adolescents use to describe their cGVHD symptoms (Wiener et al., 2014).

## Objectives and specific aims

The overall objective of this two-phase multi-center study is to develop a psychometrically valid pediatric cGVHD Symptom Scale (PCSS; including both a child and a caregiver-proxy version), as a counterpart to the LSS, to accurately and precisely capture cGVHD symptom bother in pediatric transplant survivors.

This objective will be addressed through four specific aims:

1. Evaluate the comprehensibility, clarity, acceptability, and ease of judgment of three age appropriate versions of the PCSS items and response choices with children across the developmental spectrum, and with their caregiver-proxies.
2. Evaluate the validity (concurrent validity, known-groups validity, and validity of the response scales), test–retest reliability, responsiveness, minimally important difference (MID), and the acceptability and time required to complete the PCSS (both pediatric and caregiver-proxy versions).
3. Determine the youngest age at which the PCSS can be comprehended.
4. Examine concordance between the PCSS and the LSS in order to establish the youngest age at which the LSS validly captures cGVHD symptom bother.

Guided by developmental science and expert recommendations for the development and testing of patient-reported outcome measures (Riley, 2004; Klassen et al., 2010; Mokkink et al., 2010; Patrick et al., 2011; Arbuckle and Abetz-Webb, 2013; Coyne et al., 2016; Tomlinson et al., 2016; Pinheiro et al., 2018; Terwee et al., 2018; Leahy and Steineck, 2020; Coombes et al., 2021, 2023; Rothmund et al., 2023), this multi-site study will achieve its aims in two phases. An overview of the two phases of the study is provided in Table 1.

**Table 1:** Overview of the two phases of the mixed-methods study.

Phase	Participants	Method	Inclusion criteria	Exclusion criteria	Projected sample size	Timepoints of data collection	Main objective
**Phase 1:** Content validation	Children and adolescents ages 5–17 years	Cognitive debriefing interviews	Age 5–17 years	Evidence of malignant disease relapse including molecular relapse or minimal residual disease (patients with mixed chimerism are eligible to participate)	Children age 5–7 years: *N* = 20 patient-caregiver dyads Children age 8–12 years: *N* = 20 patient-caregiver dyadsChildren age 13–17 years: *N* = 20 patient-caregiver dyads	One time interview with each patient/caregiver dyad	To use cognitive interviewing techniques to evaluate three age-appropriate versions of a new measure of cGVHD symptom bother with respect to item-level comprehension and clarity of the symptom terms, understanding of ‘symptom bother’, and the comprehension and ease of judgment of the response choices and recall period	Prior receipt of allogeneic hematopoietic stem cell transplantation
			Clinical diagnosis of cGVHD				
			Currently, receiving systemic treatment for cGVHD (including phototherapies), or has tapered systemic therapy for cGVHD to discontinuation within the past 12 months				
			English speaking				
			Absence of severe cognitive or psychiatric disability that would impair the child’s capacity for participation or completion of study related procedures, in the judgment of the investigators				
			Caregiver-proxies of participating children		Age older than 18 years				
	Caregiver-proxy of participating subject								
	Able to speak, read, write English								
	**Phase 2:** Psychometric properties		Children and adolescents ages 5–17 years	Quantitative evaluation of measurement properties (construct validity, factor structure, test–retest reliability, and responsiveness to change)	Age 5–17 yearsPrior receipt of allogeneic hematopoietic stem cell transplantationClinical diagnosis of chronic GVHD			Evidence of malignant disease relapse including molecular relapse or minimal residual disease (patients with mixed chimerism are eligible to participate)	Children age 5–7 years: *N* = 40 patient-caregiver dyads	T1 (baseline at enrollment)	Evaluate test–retest reliability, construct validity, and responsiveness of the symptom scale in association with clinical cGVHD characteristics and the Peds QL and PedsQL Stem Cell Transplant Module.			
Currently receiving systemic treatment for cGVHD (including phototherapies), or has tapered systemic therapy for cGVHD to discontinuation within the past 12 months					Children age 8–12 years: *N* = 40 patient-caregiver dyadsChildren age 13–17 years: *N* = 40 patient-caregiver dyads	T2 (24–48 h after enrollment)T3 (3 months after enrollment)	Congruence between symptom ratings provided by child participants and their caregiver-proxies will be explored.In subjects ages 13–17, we will also compare responses on the PCSS with those from the LSS, to determine the youngest age at which adolescents respondents can comprehend the LSS.				
English speaking											
Absence of severe cognitive or psychiatric disability that would impair the child’s capacity for participation or completion of study related procedures, in the judgment of the investigators								
Caregiver-proxies of participating children		Age older than 18 years						
		Caregiver-proxy of participating subject						
		Able to speak, read, and write English						

## Methods and analyses

### Phase 1: establish content validity of the candidate items through cognitive interviewing

#### Design, sample, and setting

**Figure 1:**
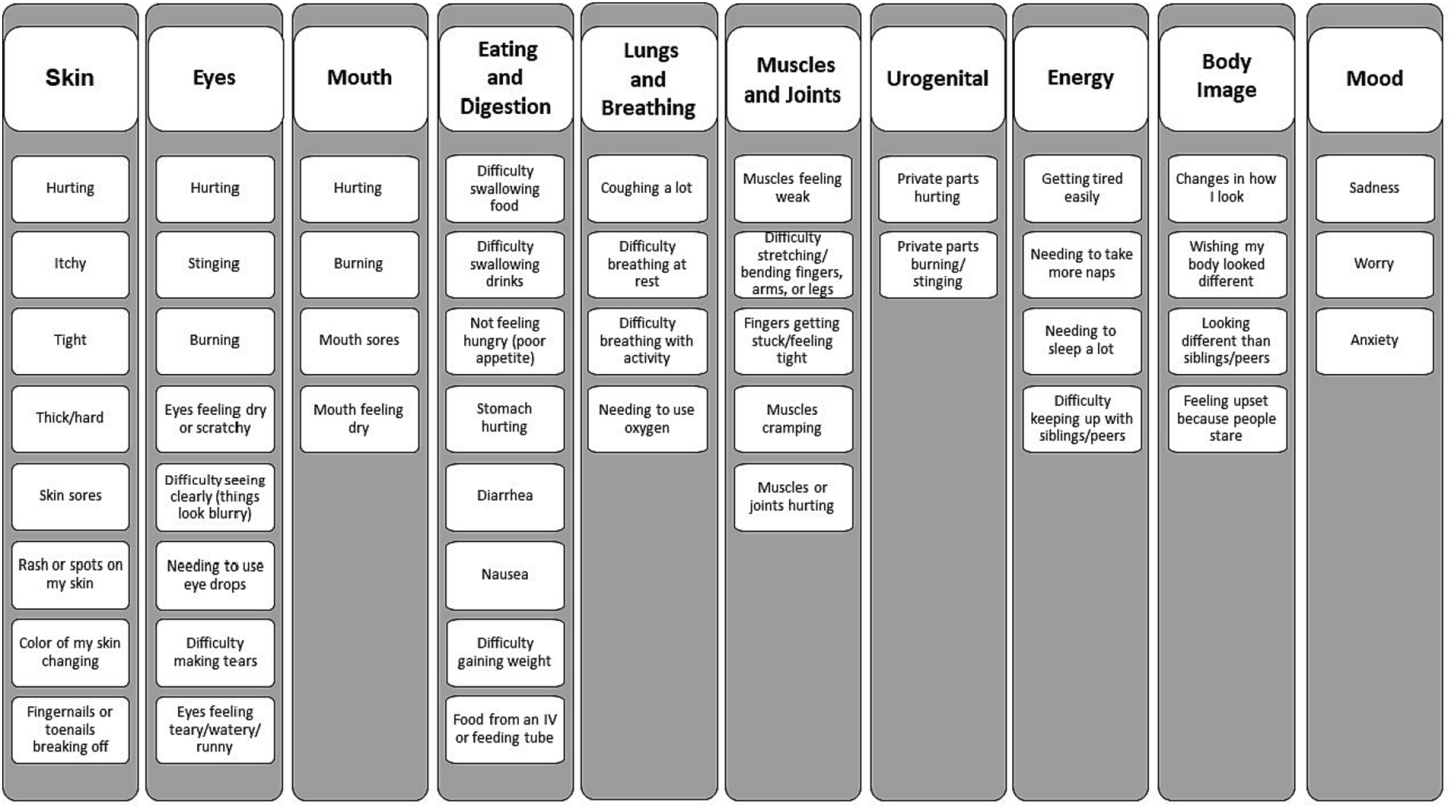
Domain framework.

A 10-domain structure (Figure 1) and an initial pool of 50 items were constructed based on our prior concept elicitation study (Wiener et al., 2014). The PCSS (both child and caregiver-proxy versions) evaluates how much the child has been bothered by each symptom in the past 1 month. The 1 month recall period and the focus on the concept of symptom bother align the PCSS with the LSS, the measure used with respondents ages 18 and older. Symptom bother incorporates symptom frequency, severity, and interference with usual activities (Gawlicki et al., 2014). Responses are provided on a three-point scale incorporating a pictogram (for respondent’s ages 5–7 years) or a five-point Likert scale (for children ages 8–17 and caregiver-proxy respondents).

Participants are recruited at one of 13 pediatric transplant centers in the United States and Canada. We will enroll three cohorts reflecting distinct developmental age bands [children ages 5–7 and their caregiver-proxies (*n* = 20 dyads); children ages 8–12 and their caregiver-proxies (*n* = 20 dyads); and children ages 13–17 and their caregiver-proxies (*n* = 20 dyads); Arbuckle and Abetz-Webb, 2013; Matza et al., 2013]. Where possible, we will attempt to balance the gender ratios within each age band. Every effort will be made to enroll participants who are currently or have recently experienced diverse clinical manifestations of cGVHD. In support of that objective, all of the children participating in the study meet established criteria for a diagnosis of cGVHD (Jagasia et al., 2015) and have received cGVHD-directed systemic therapy in the past year.

#### Overview of data collection procedures

After parent/caregiver/guardian (hereafter termed “caregiver-proxy”) has consented to participate with their child in the study, and the child assent has been obtained, a mutually convenient time is established for the interview procedures to be completed via web-based videoconferencing (Fry et al., 2021). Approximately 48 h prior to the scheduled interview, the caregiver is sent a link to complete the caregiver-proxy PCSS electronically and provide basic demographic and developmental information about their child. Subsequently a semi-scripted digitally recorded cognitive interview is conducted remotely, with the child, and then jointly with child and caregiver-proxy, to explore the comprehension, clarity and ease of response of each of the items that comprise the PCSS.

The cognitive interview schedules are tailored to children and their caregiver-proxies stratified by age (5–7, 8–12, and 13–17 years of age) and are designed to:

- Determine the extent to which each of the three age groups comprehend the symptom terms (e.g., thickened skin, tightness, and loss of appetite) and response choices of the PCSS.
- Evaluate and refine the PCSS to be clear, easy to judge, and relevant for capturing cGVHD symptom bother.
- Determine whether only caregiver-proxy report should be recommended for children in the 5–7 age range.

Cognitive interviewing is a qualitative method designed to examine the question-response process, specifically the processes and considerations used by respondents as they form answers to survey questions, and to identify and resolve sources of error (Willis, 2005). Through the interviewing process, various types of question-response problems that would not normally be identified in a traditional survey interview, such as interpretive errors and recall accuracy, are surfaced (Miller et al., 2014). In the present study, the cognitive interviews address four critical questions: (1) What is the most appropriate wording for the respondent instructions for completing the instrument (e.g., is the target concept of symptom bother well-understood)? (2) Within each age group, do respondents interpret and comprehend the symptom terms (e.g., fatigue, cutaneous symptoms, digestive symptoms, and oral and ocular symptoms) in a comparable manner? (3) Is the five-point (or in the case of 5–7 year olds, the three points) response scale well comprehended by respondents and easy for them to use to provide their responses? (4) Are the verbal descriptors (e.g., sometimes, often) used for each of the response categories clear and easy for the respondent to judge/distinguish?

#### Cognitive interviews

During the interview, the child-participant is first presented each PCSS item on the computer screen. Child-participants have each PCSS item and the response choices read to them, in addition to seeing each question and the response choices on the screen. The child provides their verbal response to the item, using the three- or five-point scale. Participants ages 8–17 are also asked whether the item was difficult to understand and/or answer. The interviewer notes any verbal or behavioral indicators of comprehension difficulties (e.g., facial expressions indicating confusion, pausing, changing an answer, and looking to the caregiver for assistance). If the child-participant requests help or clarification of any item, the interviewer encourages completion of the item to the best of their ability based on the instructions and flags that item on the interview summary.

After completing the PCSS, items identified by the child as difficult to understand, confusing or unclear and items where there were behavioral indicators of comprehension difficulties (i.e., participant appeared to be hesitant, uncertain or puzzled in making their response) are retrospectively probed. This is a common strategy in cognitive interviewing because it minimizes participants’ ability to anticipate interviewers’ questions or to change their responses to later questions which may occur if probing is conducted during the initial completion of the PRO measure. To conserve participant burden, each interview schedule is assigned a specific subset of PCSS items for probing. These items may be those symptom terms that are expected to occur less frequently (e.g., cough, shortness of breath with exercise) and/or which are anticipated to present difficulties for respondents because they reflect abstract or complex constructs/domains (e.g., genitalia, body image, muscle/joint pain, and cramping). This approach also ensures that each PCSS item receives adequate attention in the cognitive interviewing irrespective of whether the child expresses difficulties with comprehension or clarity (Arbuckle and Abetz-Webb, 2013).

After the child has been debriefed on specific items, the child and their caregiver-proxy are invited to jointly explore any differences in their responses to items in the PCSS, in order to identify whether the differing reports might reflect child non-comprehension (Tomlinson et al., 2021). By comparing the congruence between symptom reports provided by child and caregiver-proxy and probing the source of that discrepancy (i.e., misunderstanding of the question phrasing versus differences in caregiver-child appraisal of the symptom experience), we will be able to describe variation in agreement by symptom, and to explore qualitatively the basis for any divergence in ratings of symptom presence/absence provided by child-participant and caregiver-proxy. The child and caregiver-proxy will also be asked about items that should be included in the PCSS and how the existing items could be improved.

**Figure 2:**
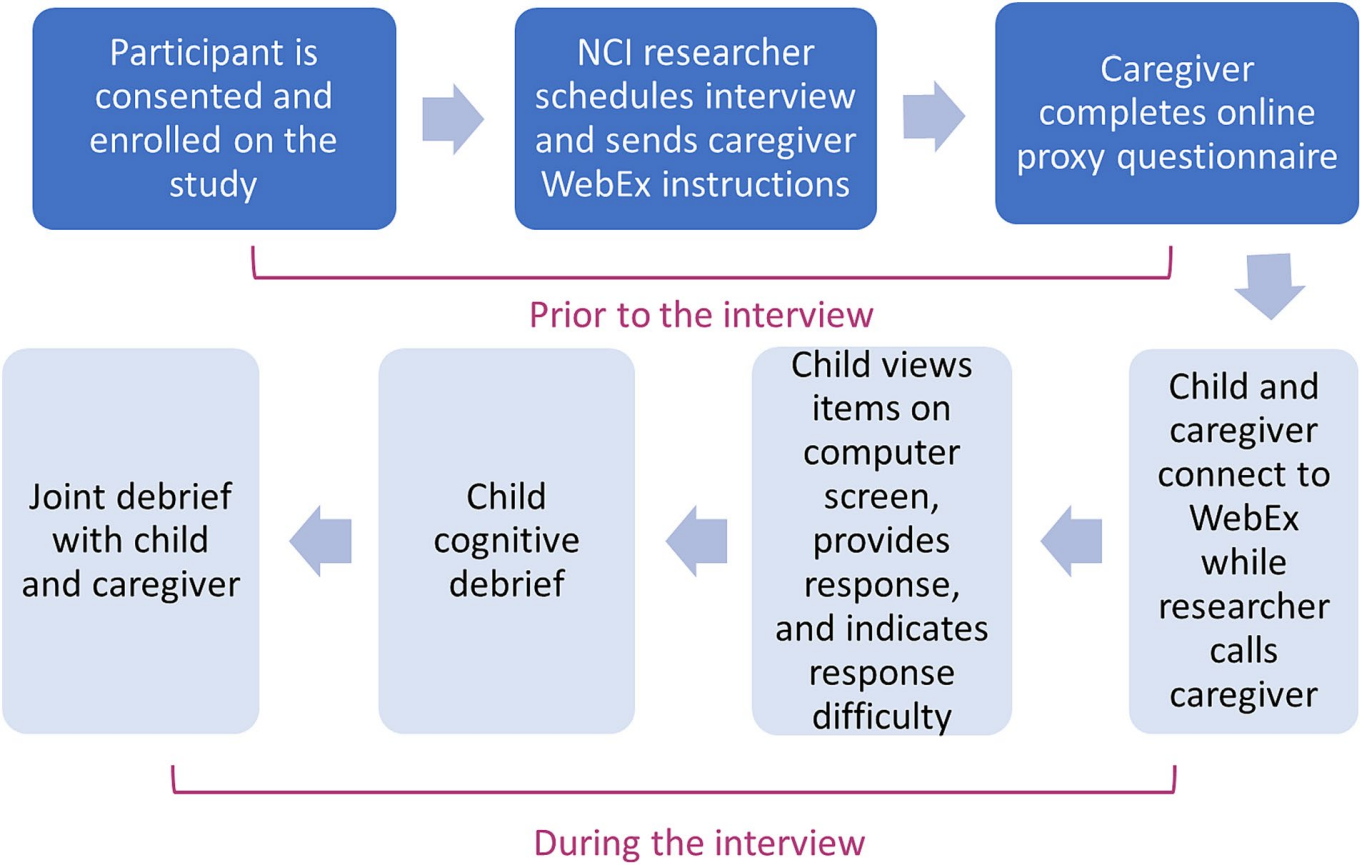
Enrollment and qualitative data collection procedures (Phase 1).

To ensure consistency in interviewing methods, interviews are centralized at the coordinating center and are performed by two trained researchers experienced with conducting pediatric qualitative interviews in medical settings. The interview and other research procedures take approximately 45–60 min. All interviews are recorded. In the case of study participants who do not have access to a computer at their home, interviews are conducted in their healthcare setting during a routine clinic appointment. Alternatively, we also offer all study measures on paper, and participants can complete interviews via telephone. Enrollment and data collection procedures are summarized in Figure 2. Table 2 describes the purpose and examples of semi-scripted interview questions.

**Table 2:** Purpose and examples of semi-scripted cognitive interview questions and probes.

Purpose of question	Examples of interview questions and prompts
Identify if items are confusing or straightforward	*How easy/difficult did you find this question to understand? How easy or difficult was it to answer? Why do you say that?*
	Solicit suggestions for rephrasing a question: *“How could the way we ask the question be improved?”*
Explore the meaning of specific words or phrases used to express the symptom terms and determine if items are comprehended, clear, and developmentally and culturally appropriate	*“What does the term [symptom term] mean to you?” “What is [symptom term]?” “What word(s) would you use to describe what [symptom term] feels like to you?” “Can you please describe in your own words what it feels like to have [symptom term]?”* Other prompts: *“If you were going to describe [symptom term] to your mom or to your friend, how would you describe it?”* *“Tell me what else you would tell your mom or your friend about [symptom term]?”* *“What happens to you when you have [symptom term]?”* *What would happen to someone who is having [symptom term]?”* *“What would you do if you have [symptom term]?”* *“How would you try to make it better” “Are there any other words that you would use that describe [symptom term] better than the word [symptom term]? If the child uses words to describe [symptom term] that are similar to other terms on the PCSS, ask “how [symptom term] is different from Y (where Y is a similar symptom on the PCSS)?”*
Determine if child understands the response options	Ages 5–7 years: Choose a common symptom that the child does not endorse as bothering them (e.g., feeling tired). Ask whether they have “*ever had this symptom* (*e.g., felt tired*).” Then choose a symptom that the child endorses as “sometimes” bothers them. Ask why they chose “*sometimes*” instead of “*bothers me a lot*” and what would have to change for the child to choose “*bothers me a lot*” over “*sometimes*.” Ask whether the child found it easy or difficult to choose between “*sometimes*” and “*a lot*”?’ Ages 8–17 years: Choose a symptom that the child does not endorse as bothering them. Ask why they said it does not bother them at all. Choose a symptom that the child endorses as “*almost never*” bothering them. Ask why they chose “*almost never*” instead of “*sometimes*.” Ask how the symptoms would have to feel to choose “*sometimes*” over “*almost never*.” Choose a symptom that the child endorsed as “*often*” bothering them and ask why they chose “*often*” vs. “*almost always*.” Ask what would have to change for the child to choose “*almost always*” for this specific symptom. Ask whether the child found it easy or difficult to choose between “*often*” and “*almost always*”? What about between “*almost never*” and “*sometimes*.” For any age group: If the child does not endorse any GVHD symptoms, ask them to remember a time when they had a cold, and tell you about something that bothered them about having a cold. Ask them if this symptom bothered them “*sometimes*” or “*almost always*.” Ask them why they chose their answer and what would have to have been different for them to choose the other response option. Ask the child if it was difficult to choose between “*sometimes*” and “*a lot*” (or for the older children, between “*sometimes*” and “*often*”).
Determine if child understands the recall period	Choose any two symptoms that the child endorses: Ask the child if they have experienced these symptoms in the past one week. Then for each symptom, ask if they experienced any of the symptoms in the past one month. Inquire how they know it was the past week only, vs. the past month or how they are sure they have had these symptoms for the past month.
Evaluate comprehension of the term “bother”	Ages 5–7 years: Ask the child what kind of things “bother” them: Ask for other words that the child uses to describe things in their life that are a “*bother*” to them. If they do not understand, provide an example. “A bellyache could bother someone so much that the bellyache makes it too hard to play with friends.” Then ask if there is another word(s) that they would use for “*bother*.” Ages 8–17 years: Ask the child what kind of things “bother” them: Ask for other words that the child uses to describe things in their life that are a “bother” to them. If they do not understand, provide an example. “A headache could bother someone so much that the headache makes it too hard to watch TV.” Then ask if there is another word(s) that would/could describe symptoms that are “bothersome.”
Explore discrepancies between the child’s and caregiver-proxy’s endorsement of a symptom as present/absent, to determine if the discrepant responses arose because of (i) child non-comprehension; (ii) child or caregiver-proxy misunderstanding of the recall period, or (iii) differences in child vs. caregiver-proxy appraisal of the symptom experience as “*bothersome*.”	Joint debriefing interview with child and caregiver-proxy: Explore discrepancies between child and caregiver-proxy symptom endorsement: Comparing the PCSS items that were endorsed by one and not by the other, say to the caregiver-proxy “*I noticed you indicated that [child] has [symptom term]. Can you tell me about this.”* Then to the child, *“I noticed you did not endorse [symptom term]. Do you agree with what your mom/dad said about you having [symptom term]? If not, can you tell me why not? If yes, what other words would you use to describe [symptom term]?”* Recall period: Ask the child about something fun they did in the past week. Then ask about anything fun they did in the past month. If they cannot recall anything fun, ask about a favorite meal they had in the past week. Then ask about a favorite meal they had in the past month. Gently inquire with the caregiver-proxy if the recollection is accurate. Additional probes: Ask the caregiver-proxy “*Is your child having any other issues that the PCSS did not ask about*?” Ask the caregiver-proxy if they thought that their child can (or did) understand item phrasing and accurately answer the questions within the PCSS Note: The caregiver-proxy will have competed the PCSS-Proxy items, and discrepancies between caregiver-proxy and child report of symptom presence/absence will have been flagged by the interviewer to prioritize items for debriefing

#### Data analysis

Following the completion of each interview, the two interviewers summarize the results for that interview. The summaries include the following information: (1) Overall themes and behavioral observations; (2) General difficulties in completing the PCSS, along with detailed descriptions of each difficulty; (3) Difficulties in answering specific questions, along with detailed descriptions of those difficulties; and (4) Responses to specific interview topics of interest [comprehension and clarity of each item, interpretation of the concept of bother, relevance of the response scale(s) and their ease of use, and understanding of the recall period]. The interviewer also notes caregiver-proxy or child recommendations for phrasing adjustments to the PCSS to improve comprehension or clarity, and documents elements of interview quality (e.g., length of the interview, any technical difficulties, child and caregiver engagement in the interview process, and relevant developmental, clinical or environmental contextual factors). The Interviewer Case Report Form (CRF; see Supplementary Table 1) was developed to code and summarize each interview. The CRF includes a place to enter the child and caregiver-proxy responses to PCSS items, capture self-reported and behavioral indicators of comprehension difficulties, summarize item-by-item comprehension as determined jointly by the two interviewers, and document the child’s verbatim statements rephrasing the item or describing its meaning in their own words that indicate comprehension or non-comprehension. While the digital recordings are not transcribed, interviewers review the recording after the interview is concluded to confirm coding or improve the capture of verbatim statements reflecting comprehension or misunderstanding.

Data are analyzed after every five interviews, and cumulative item-by-item determinations of both comprehension and saturation, stratified by age band, are documented in a coverage and saturation matrix (Kerr et al., 2010; Tomlinson et al., 2016; Meadows, 2021). Each transcript is mapped back to each PCSS item and a cumulative summary of the findings for each item and next steps (i.e., revise and retest, comparatively test a revision, continue testing, saturation achieved, and eliminate item from item pool) is constructed for each item and organized in Microsoft Excel. This approach also ensures that all PCSS items receive a comparable extent of probing across interviews within each age band and identifies the timepoint at which no new information about an item is being generated by the interviews.

Each PCSS item is debriefed with at least five children within each age band. Cognitive interviews are normally based on intensive interviewing in relatively small samples (Willis et al., 2005; Willis, 2005, 2015). Approximately 5–8 participants per item and per age group is a general rule of thumb to achieve saturation with cognitive interviews (Kerr et al., 2010; Hennink et al., 2017) since the type of logical and structural problems that we wish to identify are relatively independent of sample size (Miller et al., 2014). Symptom terms eliciting confusion or demonstrating misinterpretation by three or more respondents within an age band are considered for revision. Interview scripts are slightly refined to accommodate these changes to PCSS items.

When an item has been revised, it remains in the item pool for debriefing until it has been determined to be acceptably comprehended. Criteria or thresholds for concluding that a PCSS item is acceptably comprehended include resolution of any observed barriers to comprehension or clarity, achievement of an item-level comprehension rate (as demonstrated by the child correctly rephrasing the item in their own words) of 80% within each age band (Miller et al., 2014). An *a priori* stopping rule for concluding that comprehension of the PCSS by 5–7 year old cannot be achieved was established as the point at which 30% or more of the PCSS items are misunderstood and unable to be correctly rephrased. Study reporting will be guided by the Standards for Reporting Qualitative Research (SRQR) checklist (O'Brien et al., 2014) and the Consolidated Criteria for Reporting Qualitative Research (COREQ; Tong et al., 2007; Buus and Perron, 2020). SPSS and Excel are used to support data management and analysis.

### Phase 2: evaluate the measurement properties of the PCSS

#### Design and sample

In the second phase of this study, a cohort of 120 child and caregiver-proxy dyads, stratified by child age, will be recruited at 13 centers in the United States and Canada. Eligibility criteria are the same as those for the qualitative phase (children ages 5–17, with a diagnosis of cGVHD and currently receiving systemic immunosuppression or have had their immunosuppression tapered to discontinuation within the past 12 months). Participants in phase 1 of the study will also be approached, evaluated for eligibility, and if willing to participate, will be consented and enrolled.

#### Data collection procedures

After enrollment, the child participant and their caregiver-proxy will be provided with their own unique secure electronic link to independently complete the study measures [PCSS, PedsQL general (Varni et al., 2007c) and transplant modules (Lawitschka et al., 2014), and LSS; corresponding to their role (child or caregiver-proxy) and age band (5–7; 8–12, and 13–17 years); see Table 3 for a summary of the assessment schedule]. To examine test-re-test reliability, both the child and their caregiver-proxy will complete a second PCSS within 24–48 h of their initial completion of the measures (T2). To explore the responsiveness of the PCSS, study participants will complete the PCSS and PedsQL (general and transplant module) approximately 3 months (T3) after enrollment. They will also be asked to indicate whether their cGVHD symptoms have worsened, are unchanged, or have improved. Caregiver-proxy participants may assist their younger child (ages 5–7 and 8–12 as warranted) in completing this global assessment of change in cGVHD symptoms. Respondents ages 13–17, and their caregiver-proxies will also complete the LSS at all three timepoints. The measures are described in more detail in Supplementary Table 2.

**Table 3:** Summary of patient-reported outcomes by timing of assessment and age group (Phase 2).

Study assessments	Enrollment/Baseline	24–48 h after baseline	3 months after baseline
Child participant age 5–7 years*			
Pediatric cGVHD symptom scale (PCSS)	X	X	X
Peds QL generic module	X		X
Global self-assessment of cGVHD	X		X
**Child participant age 8–12 years**			
Pediatric cGVHD Symptom Scale (PCSS)	X	X	X
Peds QL Generic Module	X		X
Peds QL Transplant Module	X		X
Global Self-Assessment of cGVHD	X		X
**Child participant ages 13–17 years**			
Pediatric cGVHD Symptom Scale (PCSS)	X	X	X
Lee cGVHD Symptom Scale	X	X	X
Peds QL Generic Module	X		X
Peds QL Transplant Module	X		X
Global Self-Assessment of cGVHD	X		X
**Caregiver-proxy (all age groups)**			
Caregiver-proxy PCSS	X	X	X
Caregiver Peds QL Proxy	X	X	X
Caregiver Peds QL Transplant Module Proxy	X	X	X

^*^There is no child report version of the Peds QL Stem Cell Transplant Module validated for children ages 5–7. Caregivers of children ages 5–7 will complete the Caregiver Peds QL Transplant Module Proxy.

Child participants and their caregiver-proxies will receive phone and/or e-mail reminders to log into their unique survey interface to complete required study measures. To minimize missing data, completeness will be monitored prospectively by the study team. Back-up data collection procedures will be implemented for participants who do not complete their scheduled measures.

#### Statistical analysis

The primary objective of the second phase is to quantitatively evaluate the test–retest reliability, construct validity, responsiveness, and the acceptability and time required to complete the PCSS (in both pediatric and caregiver-proxy versions). STATA software will be used to support data management and analysis.

##### Reliability

For test–retest reliability, we will examine the level of agreement between total PCSS score at T1 and T2 using the Intraclass Correlation Coefficient (ICC) in all respondents, within 3–5 days of their initial completion. We will evaluate at T2 if by child and caregiver-proxy report the child remains in stable health and with stable symptoms of cGVHD (Qin et al., 2019). The ICC will be computed based on a two-way, mixed effects analysis of variance model (Shrout and Fleiss, 1979; Liljequist et al., 2019). ICC ≥ 0.70 will be interpreted as demonstrating adequate test–retest reliability.

##### Construct validity

For concurrent validity, we will examine the associations using Pearson correlation coefficients between the PCSS and the total and subscale scores of the PedsQL and PedsQL-SCTM. We will also examine associations between subscales of the PCSS and conceptually relevant items and domains of the PedsQL and the PedsQL-SCTM. We have pre-specified 25 comparisons between PCSS and the PedsQL, and PedsQL SCTM. To control for inflated Type 1 error, we will adjust for multiple comparisons using the Benjamini-Hochberg step-up method. The anticipated pooled sample size of 120 pediatric participants across the designated age cohorts (ages 5–7, 8–12, and 13–17 years) provides adequate precision for two-sided (1–0.05/25) % confidence intervals for Pearson correlation coefficients. With a sample size of 120 pediatric participants, the two-sided 99% confidence interval around a Pearson correlation of 0.8 is 0.70–0.87. Confidence interval calculations are based on a *z* transformation.

To evaluate known-groups validity, we will use independent samples *t*-tests (for two group comparisons), ANOVA (for three or more group comparisons), or Jonkheere-Terpstra tests for differences (for monotonically increasing comparison groups such as those created based on cGVHD severity scores). In evaluating known-groups validity, we will determine if mean item or mean subscale scores differ across relevant indicators of the respondent’s health status, quality of life, or clinical anchors such as NIH cGVHD severity score, NIH cGVHD organ-specific severity scores, and intensity of immunosuppression. Chi-squared tests will also be used to evaluate if the distribution of scores (range 0–4) differs between the known groups. Analysis for a clinical anchor will not be undertaken if it is observed prevalence is <20 or > 80%.

To evaluate the construct validity of the response scales, we will confirm the monotonicity of the PCSS response choices by comparing mean PedsQL total scores across subgroups with worsening PCSS item level scores using Jonckheere-Terpstra tests for differences. We will also plot mean PedsQL total scores across the response choices for each item in the PCSS. We will summarize the proportion of PCSS items where we observed monotonically decreasing mean PedsQL scores across response choices that indicate more symptom severity (higher PedsQL scores reflect better quality of life, and thus as symptom severity increases, it is expected that PedsQL scores will decline).

Given that cGVHD requiring prolonged systemic immunosuppression has an overall incidence of <20% in pediatric transplant survivors (range 5–65% incidence), we anticipate that it will prove challenging to accrue a sufficiently large sample of children to permit stratified psychometric analyses within each age cohort. Accordingly, we plan to pool data across age cohorts for the primary analysis. Sensitivity analyses will be conducted to compare results of pooled analyses with those obtained in the age-specific subgroups, thus identifying age-related trends. As described above, for the primary analysis only, *p* values will be adjusted for multiple comparisons using Benjamini-Hochberg step-up method (Keselman et al., 2002).

Prior to the primary analysis, histograms, descriptive statistics (mean, standard deviation, median, range), box plots, and or normal Q-Q plots will be reviewed for each item to identify drastic departures from normality. Data transformations and/or non-parametric methods (e.g., Wilcoxon rank sum tests and Spearman correlations) will be employed throughout should data appear highly non-normal.

We will also explore the congruence between symptom ratings provided by children and their caregiver-proxies, and examine the subscale structure using confirmatory factor analysis in children (pooling data across age cohorts to achieve a sufficiently large sample for the factor analysis). To evaluate child and caregiver-proxy congruence between symptom ratings, we will use weighted kappa with 95% confidence intervals to estimate the item-level agreement between the child’s severity rating and their caregiver-proxy’s severity rating for each symptom. Sensitivity analyses will compare the results of these pooled analyses with those derived from analyses within age cohorts, thus identifying age-related trends. Confirmatory factor analysis (CFA) will be used to explore the factor structure of the PCSS, based on the 10 domain constructs (skin, eyes, mouth, eating/digestion, lungs and breathing, muscles and joints, urogenital, energy, body image, and mood) hypothesized to underlie the PCSS. Fit indices [specifically root-mean-squared error of approximation (RMSEA), comparative fit index (CFI)] will be used to test the adequacy of the fit of the hypothesized 10-factor model to the observed data. Good model fit is indicated by RMSEA values <0.09 and/or CFI values greater than 0.90. The project sample size of 120 child respondents is likely too small to provide definitive results using CFA, however results will be informative toward the interpretability of subscale scores and will provide power estimates for a future definitive study powered to provide a robust evaluation of the PCSS factor structure.

To inform the comparability of the PCSS and the LSS which is currently used with persons aged 16 years or older, respondents between the ages of 13 and 17 will complete both measures. We will use weighted kappa to estimate agreement between PCSS responses and responses to corresponding items from the LSS, in children between the ages of 13 and 17 and their caregiver-proxies. While this does not substitute for a full psychometric test of the equivalence of both versions, results will provide initial empiric estimates of the lowest age at which younger adolescents can meaningfully complete the LSS, and preliminary information about the comparability of PCSS and LSS values which may be useful toward justifying pooled analyses or interpreting outcomes specifically in trials of new cGVHD therapies when both the PCSS and the LSS are used. In subgroup analysis of respondents ages 13–17, we will examine the congruence between their symptom endorsement rates and their symptom severity ratings on the items of the PCSS and corresponding items in the LSS. This will inform our knowledge of the comparability of both measures in younger and older adolescents and derive preliminary estimates of the lowest age at which young adolescents can meaningfully complete the LSS.

##### Responsiveness to change

To explore the responsiveness of the PCSS, study participants will be categorized into three groups based on responses to a question about their global impression of symptom change from baseline to 3 months (Coon and Cook, 2018). Within each group (no change, worsened, and improved), the total PCSS score will be compared used a paired *t*-test. The standardized response mean will be computed as the mean change scored divided by the standard deviation of the change scores within each change category (worsening vs. no change vs. improvement). Values greater than 0.8 will be considered large and values between 0.5 and 0.8 will be considered moderate. Subsequently the change in total score will be investigated using analysis of covariance in which the T1 score is a covariate and change in symptom severity (worsening vs. no change vs. improvement) is a factor in modeling the T2 rating. Lastly, change scores will be modeled using a generalized linear regression model. cGVHD severity scores and change in PedsQL physical function scores will be incorporated into this model in confirmatory analysis. These various approaches to examining responsiveness to change will be triangulated to preliminarily propose clinically meaningful thresholds for change at the individual and group level (Trigg and Griffiths, 2021).

## Ethical, equity, and regulatory considerations

Children of both genders and from all racial and ethnic groups are eligible for this study if they meet the eligibility criteria outlined in the study protocol. Children under the age of 5 are excluded because these children are unlikely to be able to understand and report on the concepts of symptom intensity and interference (Tomlinson et al., 2021), especially for symptoms such as muscle cramping, skin thickening, itching, shortness of breath, muscle/joint pain, and body image changes all of which are common in cGVHD. Non-English speaking participants are not included because at this initial stage of measure validation, it is important to first create a reliable and valid measure in English before it is put through the rigorous translation and linguistic validation process used to create versions of the measure in other languages. A finalized English language measure will not be available until after Phase 2 is completed; as such, this work provides the foundation for future studies to develop and test the PCSS for children and caregiver-proxies who speak languages other than English. Several design features of this multi-site study including representation from different geographic regions of United States and Canada and the use of remote interviewing techniques support our efforts to recruit a diverse population with respect to gender, race and ethnicity, socioeconomic status, and severity of cGVHD (Upadhyay and Lipkovich, 2020).

Parent/caregiver consent and child participant assent is obtained from each family. Where deemed appropriate by the consenting site investigator and the child’s parent(s) or guardian, the child will also be included in all discussions about the trial, and age-appropriate language will be used to describe the procedures and tests involved in this study, along with the risks, discomforts, and benefits of participation. The assent process will take place in conjunction with consent; therefore, in person and remote assent are permitted under the same circumstances as in person and remote consent. Children under 18 will provide assent for their participation in accordance with their institution’s local requirements and the assent process will be documented per local procedures. A waiver of the documentation of informed consent for caregivers of participants who will be completing questionnaires about their children and participating in cognitive interviews was granted by the IRB at the coordinating center.

Throughout the study, participants may be asked to discuss issues that are sensitive such as body image changes, and mood, and could experience distress as a result. To address this concern, study team members explain during the consent process that should this occur, an option to follow-up with a clinical team member will be provided.

Participant information is coded with an ID number. Project data will be stored in a password protected secure computing environment to ensure that only approved members of the research team will have access to these data.

## Discussion

There is currently no available pediatric PRO measure of cGVHD symptom bother. Building upon our prior concept elicitation work (Wiener et al., 2014), this multi-center two-phase study uses quantitative and qualitative methods to develop, refine, and establish the measurement properties of a new pediatric measure of cGVHD symptom bother for children and their caregiver-proxies, the Pediatric cGVHD Symptom Scale or PCSS. In phase 1, we will use cognitive interviewing to examine and refine the comprehensibility, clarity and ease of response of the PCSS. In phase 2, we will use classical and modern measurement theory to examine the test–retest reliability, construct validity, and responsiveness to change of this new measure. Knowledge derived from this study will contribute to refinement of the item phrasing across diverse age groups, provide evidence that the instrument has acceptable preliminary measurement properties to support its use in clinical trials and clinical practice, and offer preliminary estimates of clinically important change that improve its interpretability as an outcome measure.

Several design features contribute to the strengths of this study including sample recruitment at 13 geographically dispersed transplant centers in the United States and Canada, which permits enrollment of a diverse sample with respect to age bands, cGVHD manifestations, race/ethnicity, and other sociodemographic factors. Inclusion of respondents currently receiving systemic treatment for cGVHD and those who have recently tapered systemic immunosuppression to discontinuation will mitigate the possibility that the reliability and validity estimates are inflated by the absence of symptoms. Our study design also includes several different objective measures of cGVHD manifestations and their clinical severity to strengthen interpretations of known groups validity, clinical stability (for test–retest reliability), and clinically significant change over time (Paiva et al., 2014). The study adheres to international consensus standards for the development, refinement, and validation of pediatric PRO measures (Patrick et al., 2011; Arbuckle and Abetz-Webb, 2013; Matza et al., 2013; Reeve et al., 2013).

The study has some potential caveats that will need to be considered in interpreting the findings and that should be addressed in future research. First, while the sample sizes generally accord with recommended guidelines for cognitive interviewing studies to establish content validity (Willis, 2005; DeWalt et al., 2007; Patrick et al., 2011; Miller et al., 2014), recruitment of a larger sample of children with cGVHD, which is a relatively rare complication, would increase rigor yet would also be logistically challenging to achieve. In addition, while dividing the sample into narrow age bands is consistent with expert recommendations for development of a pediatric PRO and helps to assure developmentally appropriate item phrasing (Arbuckle and Abetz-Webb, 2013), it could also be the case that important findings within any one age group might be missed due to sparse data. At the same time, in both phases of our study, the interpretability of our findings is strengthened by the fact that study participants’ clinical characteristics are similar to those of the children who will complete this symptom scale in the future. Further, the sample population for this two-phase study may not ultimately be representative of the entire cGVHD patient population since only English-speaking respondents are included. We also excluded children with significant cognitive disabilities as we wanted to distinguish between children who did not understand a PCSS item due to their disability vs. a poorly worded item. As such, future studies may need to enhance recruitment of children with disease- and/or treatment-related cognitive deficits, and children and caregiver-proxies with lower English proficiency. Instrument validation is an iterative process that includes both quantitative and qualitative methods to evaluate and strengthen the measurement properties of an instrument. Continued research to confirm and extend the insights derived from this study will be required. Despite these limitations, the results that are anticipated after conducting this two phase study are expected to be useful in helping children to self-report bothersome cGVHD symptoms that can negatively affect their daily life.

## Conclusion

This study constitutes an important first step toward our long-term research goal of making available a psychometrically sound pediatric PRO measure of cGVHD symptoms. This new measure will serve as a companion to the Lee cGVHD Symptom Scale used with individuals older than age 18 that can be integrated into clinical trials and care delivery for pediatric allogeneic hematopoietic stem cell transplant survivors.

## Ethics statement

All study participants provided consent to participate in this study.

## Author contributions

SM and LW: study conception and design. SM, LW, RH, AF, and SP: participant recruitment and data collection. SM, LW, RH, AF, SP, and BW: analysis and interpretation of data and drafting of the manuscript. All authors contributed to the article and approved the submitted version.

## References

[ref1] Agh T. Csanadi M. Voko Z. Webb T. Jeyakumaran D. Trudeau J. . (2019). Humanistic burden of patients with chronic graft-versus-host disease—systematic literature review of health-related quality of life and functional status. Expert. Rev. Hematol. 12, 295–309. doi: 10.1080/17474086.2019.1602036, PMID: 30925855

[ref2] Arbuckle R. Abetz-Webb L. (2013). "not just little adults": qualitative methods to support the development of pediatric patient-reported outcomes. Patient 6, 143–159. doi: 10.1007/s40271-013-0022-3 23912695

[ref3] Bele S. Chugh A. Mohamed B. Teela L. Haverman L. Santana M. J. (2020). Patient-reported outcome measures in routine pediatric clinical care: a systematic review. Front. Pediatr. 8:364. doi: 10.3389/fped.2020.00364, PMID: 32850521 PMC7399166

[ref4] Buus N. Perron A. (2020). The quality of quality criteria: replicating the development of the consolidated criteria for reporting qualitative research (COREQ). Int. J. Nurs. Stud. 102:103452. doi: 10.1016/j.ijnurstu.2019.103452, PMID: 31726311

[ref5] Carpenter P. A. Kitko C. L. Elad S. Flowers M. E. Gea-Banacloche J. C. Halter J. P. . (2015). National Institutes of Health consensus development project on criteria for clinical trials in chronic graft-versus-host disease: V. The 2014 ancillary therapy and supportive care working group report. Biol. Blood Marrow Transplant. 21, 1167–1187. doi: 10.1016/j.bbmt.2015.03.024, PMID: 25838185 PMC4821166

[ref6] Coombes L. Bristowe K. Ellis-Smith C. Aworinde J. Fraser L. K. Downing J. . (2021). Enhancing validity, reliability and participation in self-reported health outcome measurement for children and young people: a systematic review of recall period, response scale format, and administration modality. Qual. Life Res. 30, 1803–1832. doi: 10.1007/s11136-021-02814-4, PMID: 33738710 PMC8233251

[ref7] Coombes L. Harðardóttir D. Braybrook D. Roach A. Scott H. Bristowe K. . (2023). Design and Administration of Patient-Centred Outcome Measures: the perspectives of children and young people with life-limiting or life-threatening conditions and their family members. Patient 16, 473–483. doi: 10.1007/s40271-023-00627-w, PMID: 37221441 PMC10205035

[ref8] Coon C. D. Cook K. F. (2018). Moving from significance to real-world meaning: methods for interpreting change in clinical outcome assessment scores. Qual. Life Res. 27, 33–40. doi: 10.1007/s11136-017-1616-3 28620874

[ref9] Coyne I. Amory A. Gibson F. Kiernan G. (2016). Information-sharing between healthcare professionals, parents and children with cancer: more than a matter of information exchange. Eur. J. Cancer Care 25, 141–156. doi: 10.1111/ecc.12411, PMID: 26537295

[ref10] Cuvelier G. D. E. Nemecek E. R. Wahlstrom J. T. Kitko C. L. Lewis V. A. Schechter T. . (2019). Benefits and challenges with diagnosing chronic and late acute GVHD in children using the NIH consensus criteria. Blood 134, 304–316. doi: 10.1182/blood.2019000216, PMID: 31043425 PMC6911839

[ref11] DeWalt D. A. Rothrock N. Yount S. Stone A. A. (2007). Evaluation of item candidates: the PROMIS qualitative item review. Med. Care 45, S12–S21. doi: 10.1097/01.mlr.0000254567.79743.e200005650-200705001-00003[pii], PMID: 17443114 PMC2810630

[ref12] Fry A. Mitchell S. A. Wiener L. (2021). Considerations for conducting and reporting digitally supported cognitive interviews with children and adults. J. Patient Rep. Outcomes 5:131. doi: 10.1186/s41687-021-00371-5, PMID: 34921668 PMC8683807

[ref13] Gawlicki M. C. McKown S. M. Talbert M. J. Brandt B. A. (2014). Application of bother in patient reported outcomes instruments across cultures. Health Qual. Life Outcomes 12:18. doi: 10.1186/1477-7525-12-18, PMID: 24520950 PMC3927629

[ref14] Haroun E. Agrawal K. Leibovitch J. Kassab J. Zoghbi M. Dutta D. . (2023). Chronic graft-versus-host disease in pediatric patients: differences and challenges. Blood Rev. 60:101054. doi: 10.1016/j.blre.2023.101054, PMID: 36805299

[ref15] Hennink M. M. Kaiser B. N. Marconi V. C. (2017). Code saturation versus meaning saturation: how many interviews are enough? Qual. Health Res. 27, 591–608. doi: 10.1177/1049732316665344, PMID: 27670770 PMC9359070

[ref16] Jagasia M. H. Greinix H. T. Arora M. Williams K. M. Wolff D. Cowen E. W. . (2015). National Institutes of Health consensus development project on criteria for clinical trials in chronic graft-versus-host disease: I. The 2014 diagnosis and staging working group report. Biol. Blood Marrow Transplant. 21, 389–401. doi: 10.1016/j.bbmt.2014.12.001 25529383 PMC4329079

[ref17] Kerr C. Nixon A. Wild D. (2010). Assessing and demonstrating data saturation in qualitative inquiry supporting patient-reported outcomes research. Expert Rev. Pharmacoecon. Outcomes Res. 10, 269–281. doi: 10.1586/erp.10.30, PMID: 20545592

[ref18] Keselman H. J. Cribbie R. Holland B. (2002). Controlling the rate of type I error over a large set of statistical tests. Br. J. Math. Stat. Psychol. 55, 27–39. doi: 10.1348/000711002159680 12034010

[ref19] Kitko C. L. Pidala J. Schoemans H. M. Lawitschka A. Flowers M. E. Cowen E. W. . (2021). National Institutes of Health consensus development project on criteria for clinical trials in chronic graft-versus-host disease: IIa. The 2020 clinical implementation and early diagnosis working group report. Transplant Cell Ther. 27, 545–557. doi: 10.1016/j.jtct.2021.03.033, PMID: 33839317 PMC8803210

[ref20] Klassen A. F. Strohm S. J. Maurice-Stam H. Grootenhuis M. A. (2010). Quality of life questionnaires for children with cancer and childhood cancer survivors: a review of the development of available measures. Support Care Cancer 18, 1207–1217. doi: 10.1007/s00520-009-0751-y, PMID: 19834745

[ref21] Lawitschka A. Buehrer S. Bauer D. Peters K. Silbernagl M. Zubarovskaya N. . (2020). A web-based Mobile app (INTERACCT app) for adolescents undergoing Cancer and hematopoietic stem cell transplantation aftercare to improve the quality of medical information for clinicians: observational study. JMIR Mhealth Uhealth 8:e18781. doi: 10.2196/18781, PMID: 32602847 PMC7367529

[ref22] Lawitschka A. Guclu E. D. Varni J. W. Putz M. Wolff D. Pavletic S. . (2014). Health-related quality of life in pediatric patients after allogeneic SCT: development of the PedsQL stem cell transplant module and results of a pilot study. Bone Marrow Transplant. 49, 1093–1097. doi: 10.1038/bmt.2014.96, PMID: 24820217

[ref23] Leahy A. B. Steineck A. (2020). Patient-reported outcomes in pediatric oncology: the patient voice as a gold standard. JAMA Pediatr. 174:e202868. doi: 10.1001/jamapediatrics.2020.2868, PMID: 32832974 PMC8103813

[ref24] Lee S. Cook E. F. Soiffer R. Antin J. H. (2002). Development and validation of a scale to measure symptoms of chronic graft-versus-host disease. Biol. Blood Marrow Transplant. 8, 444–452. doi: 10.1053/bbmt.2002.v8.pm12234170, PMID: 12234170

[ref25] Lee S. J. Cutler C. Blazar B. R. Tu A. Yang Z. Pavletic S. Z. (2022). Correlation of patient-reported outcomes with clinical organ responses: data from the Belumosudil chronic graft-versus-host disease studies. Transplant Cell Ther. 28, 700.e1–700.e6. doi: 10.1016/j.jtct.2022.06.020, PMID: 35781099 PMC10634740

[ref26] Lee S. J. Onstad L. Chow E. J. Shaw B. E. Jim H. S. L. Syrjala K. L. . (2018). Patient-reported outcomes and health status associated with chronic graft-versus-host disease. Haematologica 103, 1535–1541. doi: 10.3324/haematol.2018.192930, PMID: 29858386 PMC6119141

[ref27] Lee S. J. Wolff D. Kitko C. Koreth J. Inamoto Y. Jagasia M. . (2015). Measuring therapeutic response in chronic graft-versus-host disease. National Institutes of Health consensus development project on criteria for clinical trials in chronic graft-versus-host disease: IV. The 2014 response criteria working group report. Biol. Blood Marrow Transplant. 21, 984–999. doi: 10.1016/j.bbmt.2015.02.025, PMID: 25796139 PMC4744804

[ref28] Liljequist D. Elfving B. Skavberg Roaldsen K. (2019). Intraclass correlation—a discussion and demonstration of basic features. PLoS One 14:e0219854. doi: 10.1371/journal.pone.0219854, PMID: 31329615 PMC6645485

[ref29] Matza L. S. Patrick D. L. Riley A. W. Alexander J. J. Rajmil L. Pleil A. M. . (2013). Pediatric patient-reported outcome instruments for research to support medical product labeling: report of the ISPOR PRO good research practices for the assessment of children and adolescents task force. Value Health 16, 461–479. doi: 10.1016/j.jval.2013.04.004, PMID: 23796280

[ref30] Meadows K. (2021). Cognitive interviewing methodologies. Clin. Nurs. Res. 30, 375–379. doi: 10.1177/10547738211014099 33998325

[ref31] Merkel E. C. Mitchell S. A. Lee S. J. (2016). Content validity of the Lee chronic graft-versus-host disease symptom scale as assessed by cognitive interviews. Biol. Blood Marrow Transplant. 22, 752–758. doi: 10.1016/j.bbmt.2015.12.026, PMID: 26751003 PMC4850024

[ref32] Miller K. Willson S. Chepp V. Padilla J. (eds.). (2014). Cognitive Interviewing Methodology. Hoboken, NJ: John Wiley & Sons

[ref33] Mitchell S. A. Leidy N. K. Mooney K. H. Dudley W. N. Beck S. L. LaStayo P. C. . (2010). Determinants of functional performance in long-term survivors of allogeneic hematopoietic stem cell transplantation with chronic graft-versus-host disease (cGVHD). Bone Marrow Transplant. 45, 762–769. doi: 10.1038/bmt.2009.238, PMID: 19784078 PMC2850962

[ref34] Mitchell S. A. Wiener L. Hoag J. Fry A. Bevans M. F. (2021). “Health-related quality of life in adult and pediatric survivors” in Blood and Marrow Transplantation Long Term Management. eds. Savani B. Tichelli A.. 2nd ed (Hoboken, NJ: Wiley-Blackwell), 355–380.

[ref35] Mokkink L. B. Terwee C. B. Patrick D. L. Alonso J. Stratford P. W. Knol D. L. . (2010). The COSMIN study reached international consensus on taxonomy, terminology, and definitions of measurement properties for health-related patient-reported outcomes. J. Clin. Epidemiol. 63, 737–745. doi: 10.1016/j.jclinepi.2010.02.006, PMID: 20494804

[ref36] O'Brien B. C. Harris I. B. Beckman T. J. Reed D. A. Cook D. A. (2014). Standards for reporting qualitative research: a synthesis of recommendations. Acad. Med. 89, 1245–1251. doi: 10.1097/acm.0000000000000388 24979285

[ref37] Paiva C. E. Barroso E. M. Carneseca E. C. de Pádua Souza C. Dos Santos F. T. Mendoza López R. V. . (2014). A critical analysis of test-retest reliability in instrument validation studies of cancer patients under palliative care: a systematic review. BMC Med. Res. Methodol. 14:8. doi: 10.1186/1471-2288-14-8, PMID: 24447633 PMC3899385

[ref38] Patrick D. L. Burke L. B. Gwaltney C. J. Leidy N. K. Martin M. L. Molsen E. . (2011). Content validity--establishing and reporting the evidence in newly developed patient-reported outcomes (PRO) instruments for medical product evaluation: ISPOR PRO good research practices task force report: part 2--assessing respondent understanding. Value Health 14, 978–988. doi: 10.1016/j.jval.2011.06.013 22152166

[ref39] Pinheiro L. C. McFatrich M. Lucas N. Walker J. S. Withycombe J. S. Hinds P. S. . (2018). Child and adolescent self-report symptom measurement in pediatric oncology research: a systematic literature review. Qual. Life Res. 27, 291–319. doi: 10.1007/s11136-017-1692-4, PMID: 28879501 PMC5823735

[ref40] Qin S. Nelson L. McLeod L. Eremenco S. Coons S. J. (2019). Assessing test-retest reliability of patient-reported outcome measures using intraclass correlation coefficients: recommendations for selecting and documenting the analytical formula. Qual. Life Res. 28, 1029–1033. doi: 10.1007/s11136-018-2076-0, PMID: 30547346 PMC6439259

[ref41] Reeve B. B. Wyrwich K. W. Wu A. W. Velikova G. Terwee C. B. Snyder C. F. . (2013). ISOQOL recommends minimum standards for patient-reported outcome measures used in patient-centered outcomes and comparative effectiveness research. Qual. Life Res. 22, 1889–1905. doi: 10.1007/s11136-012-0344-y, PMID: 23288613

[ref42] Riley A. W. (2004). Evidence that school-age children can self-report on their health. Ambul. Pediatr. 4, 371–376. doi: 10.1367/a03-178r.1 15264962

[ref43] Rothmund M. Meryk A. Rumpold G. Crazzolara R. Sodergren S. Darlington A. S. . (2023). A critical evaluation of the content validity of patient-reported outcome measures assessing health-related quality of life in children with cancer: a systematic review. J. Patient Rep. Outcomes 7:2. doi: 10.1186/s41687-023-00540-8, PMID: 36656407 PMC9851583

[ref44] Shrout P. Fleiss J. (1979). Intraclass correlations: uses in assessing rater reliability. Psychol. Bull. 86, 420–428. doi: 10.1037//0033-2909.86.2.420 18839484

[ref45] Terwee C. B. Prinsen C. A. C. Chiarotto A. Westerman M. J. Patrick D. L. Alonso J. . (2018). COSMIN methodology for evaluating the content validity of patient-reported outcome measures: a Delphi study. Qual. Life Res. 27, 1159–1170. doi: 10.1007/s11136-018-1829-0, PMID: 29550964 PMC5891557

[ref46] Tomlinson D. De Mol Van Otterloo S. O'Sullivan C. Gibson P. Johnston D. L. Portwine C. . (2016). Methodological issues identified during cognitive interviews in the development of a pediatric cancer symptom screening tool. Psychooncology 25, 349–353. doi: 10.1002/pon.3821, PMID: 25920596

[ref47] Tomlinson D. Plenert E. Dadzie G. Loves R. Cook S. Schechter T. . (2021). Reasons for disagreement between proxy-report and self-report rating of symptoms in children receiving cancer therapies. Support Care Cancer 29, 4165–4170. doi: 10.1007/s00520-020-05930-y, PMID: 33404808

[ref48] Tong A. Sainsbury P. Craig J. (2007). Consolidated criteria for reporting qualitative research (COREQ): a 32-item checklist for interviews and focus groups. Int. J. Qual. Health Care 19, 349–357. doi: 10.1093/intqhc/mzm042, PMID: 17872937

[ref49] Trigg A. Griffiths P. (2021). Triangulation of multiple meaningful change thresholds for patient-reported outcome scores. Qual. Life Res. 30, 2755–2764. doi: 10.1007/s11136-021-02957-4, PMID: 34319532

[ref50] Upadhyay U. D. Lipkovich H. (2020). Using online technologies to improve diversity and inclusion in cognitive interviews with young people. BMC Med. Res. Methodol. 20:159. doi: 10.1186/s12874-020-01024-9, PMID: 32539726 PMC7295690

[ref51] Varni J. W. Burwinkle T. M. Katz E. R. Meeske K. Dickinson P. (2002). The PedsQL (TM) in pediatric cancer—reliability and validity of the pediatric quality of life inventory (TM) generic Core scales, multidimensional fatigue scale, and Cancer module. Cancer 94, 2090–2106. doi: 10.1002/Cncr.10427 11932914

[ref52] Varni J. W. Burwinkle T. M. Seid M. Skarr D. (2003). The PedsQL (TM) 4.0 as a pediatric population health measure: feasibility, reliability, and validity. Ambul. Pediatr. 3, 329–341. doi: 10.1367/1539-4409(2003)003<0329:Tpaapp>2.0.Co;2 14616041

[ref53] Varni J. W. Katz E. R. Seid M. Quiggins D. J. L. Friedman-Bender A. (1998). The pediatric cancer quality of life inventory-32 (PCQL-32) – I. Reliability and validity. Cancer 82, 1184–1196. doi: 10.1002/(Sici)1097-0142(19980315)82:6<1184::Aid-Cncr25>3.0.Co;2-1, PMID: 9506367

[ref54] Varni J. W. Limbers C. A. (2009). The PedsQL (TM) 4.0 generic Core scales young adult version feasibility, reliability and validity in a university student population. J. Health Psychol. 14, 611–622. doi: 10.1177/1359105309103580, PMID: 19383661

[ref55] Varni J. W. Limbers C. Burwinkle T. M. (2007a). Literature review: health-related quality of life measurement in pediatric oncology: hearing the voices of the children. J. Pediatr. Psychol. 32, 1151–1163. doi: 10.1093/jpepsy/jsm008, PMID: 17347186

[ref56] Varni J. W. Limbers C. A. Burwinkle T. M. (2007b). How young can children reliably and validly self-report their health-related quality of life?: an analysis of 8,591 children across age subgroups with the PedsQL 4.0 generic Core scales. Health Qual. Life Outcomes 5:1. doi: 10.1186/1477-7525-5-1, PMID: 17201920 PMC1769360

[ref57] Varni J. W. Limbers C. A. Burwinkle T. M. (2007c). Parent proxy-report of their children's health-related quality of life: an analysis of 13,878 parents' reliability and validity across age subgroups using the PedsQL (TM) 4.0 generic Core scales. Health Qual. Life Outcomes 5, 1–10. doi: 10.1186/1477-7525-5-2 17201923 PMC1769359

[ref58] Wiener L. Baird K. Crum C. Powers K. Carpenter P. Baker K. S. . (2014). Child and parent perspectives of the chronic graft-versus-host disease (cGVHD) symptom experience: a concept elicitation study. Support Care Cancer 22, 295–305. doi: 10.1007/s00520-013-1957-6, PMID: 24077685 PMC5176341

[ref59] Willis G. B. (2005). Cognitive Interviewing: A Tool for Improving Questionnaire Design. Thousand Oaks, CA: SAGE Publications, Inc

[ref60] Willis G. B. (2015). The practice of cross-cultural cognitive interviewing. Public Opin. Q. 79, 359–395. doi: 10.1093/poq/nfu092

[ref61] Willis G. B. Reeve B. B. Barofsky I. (2005). “The use of cognitive interviewing techniques in quality of life and patient-reported outcomes measurement” in Outcomes Assessment in Cancer: Findings and Recommendations of the Cancer Outcomes Measurement Working Group. eds. Lipscomb J. Gotay C. C. Snyder C. (Cambridge, UK: Cambridge University Press), 610–622.

