# Supplementary material for: Development and psychometric testing of a pediatric chronic graft-versus-host disease symptom scale: protocol for a two-phase, mixed methods study
Source: Front Psychol. 2024 Jan 8;14:1243005. doi: 10.3389/fpsyg.2023.1243005 (PMC10800914; doi:10.3389/fpsyg.2023.1243005)
Supplement: Supplementary file 1 [file Data_Sheet_1.docx]

**Supplemental Table 1:** Example of Interviewer Case Report Form

| **PCSS Symptom Term**  **I am/my child is bothered by…** | **Child Response**  **(0-4 scale)** | **Caregiver- Proxy Response**  **(0-4 scale )** | **Self-Reported Indicator of Non-Comprehension (Yes/No)**  (Child indicates item was difficult to understand and/or answer) | **Behavioral Indicator of Non-Comprehension (Yes/No)** | **Behavioral Indicator of Non-Comprehension (Describe)** | **Child able to rephrase the meaning of the item in their own words**  **(Yes/No)** | **Notes/Quotes from Debriefing Interview with Child and Joint Debrief with Child and Caregiver-Proxy** | **Item-Level Summary Determination of Comprehension** |
| --- | --- | --- | --- | --- | --- | --- | --- | --- |
| Skin feeling itchy | 2 | 0 | No | No | Not applicable | Yes | Child: “Sometimes an itch feels like a mosquito just bit me, but I just put on the [medicated] special cream I have so hasn’t bothered me enough to tell my mom”  Caregiver-Proxy: “I haven’t heard her complain about itching much in the past one month” | Comprehended |
| Eyes feeling dry or scratchy | 0 | 2 | N | No | Not applicable | No | Child: “I’m not sure what is meant by scratchy … sometimes I have trouble seeing faraway and I rub my eyes a lot”  Field Note: Despite not endorsing difficulty with comprehension, child was unable to rephrase in their own words, and confused dry or scratchy eyes with trouble seeing/blurry vision | Child did not comprehend |
| Looking different than my friends, siblings, or classmates | 3 | 0 | No | Yes | Child had hesitation and a long pause before providing their answer, and after providing answer looked at caregiver-proxy for validation | Yes | Child: “I don’t like talking about it, but yes sometimes knowing how weird I know I look makes me very sad”  Caregiver-Proxy: “I had no idea how uncomfortable he is with his appearance and I am sad for him too and wish that there was something that could be done”  Field Note: Interviewers attributed child’s hesitation and pause to discomfort answering questions about this topic, similar patterns were observed when debriefing other “appearance” questions | Comprehended |

**Supplemental Table 2:** Description of Phase 2 Measures

| **Instrument** | **Description** | **Measurement Properties** |
| --- | --- | --- |
| Pediatric Quality of Life (PedsQL™) Inventory Version 4.0 Short Forms (SF15) for child self report ages 5-18, and parent-proxy report (Varni et al., 1998; Varni et al., 2002; Varni et al., 2003; Varni et al., 2007a; Varni et al., 2007c; b; Varni and Limbers, 2009) | - The PedsQL 4.0 Short Forms. Generic Core Scales (child versions are matched to age bands) comprise 15 items assessing functioning across 4 domains: physical (5 items), emotional (4 items), social (3 items), and school (3 items). - The parent version of the PedsQL evaluates domains comparable to those in the child version. - Response Choices: 5-point Likert scale ranging from 0 "Never a problem" to 4 "Almost always a problem". Response choices for ages 5-7: 3-point scale with the response options 0 "not at all a problem"; 2 "sometimes a problem" and 3 "a lot of a problem". - Recall period: Past 7 days - For ease of interpretability, items are reversed scored and linearly transformed to a 0-100 scale, so that higher scores indicate better health-related quality of life. - Responses from the emotional, social, and school functioning scales comprise the Psychosocial Health Summary Score. The Physical Health Summary Score is the same as the physical functioning subscale scores. All items combine to produce a total health-related quality of life score. - In addition to a total score, an “at risk” cut-off score of ≤ 69.71 has been suggested by the PedsQL developers. Children scoring at or below this cut-off value have quality of life similar to that of chronically ill children. | - Reliability: Alpha coefficients for the generic core scales in both child and parent proxy-report were >0.70, and for the full 23-item scale were 0.90 for self- and proxy-report. - Construct validity: Moderate to strong correlations with other measures of disease burden were seen. Child- and proxy-report distinguished between children with and without a chronic health condition, and within the group of children with a chronic condition, between those who did or did not have an overnight hospital visit in the last 12 months. Both child self-report and parent proxy-report correlated significantly with the number of days the child was too ill to pursue normal activities, needed someone to care for them, missed school in the last month, the number of days the parent missed from work in the last month, and parent-report of problems pursuing their normal work routine and concentrating at work. The PedsQL Generic Core Scales have also demonstrated responsiveness to clinical change. |
| Pediatric Quality of Life Stem Cell Transplant Module – Self-report and Parent-proxy (PedsQL-SCTM) [Acute Version 1.0] for child self report ages 8-18 and parent-proxy report (Lawitschka et al., 2014; Lawitschka et al., 2020) | - PedsQL Stem Cell Transplant module consists of 39 - 41 items (varies by age group) addressing several transplant relevant health-related quality of life domains: pain, fatigue, sleep, weakness, nausea, worry/anxiety about disease/treatment, nutritional problems, neurocognitive problems, communication about disease/treatment, loneliness, physical functioning and additional somatic complaints (pruritus, skin inflammation, oral problems, eyes or breathing) - Response choices: 5-point Likert-type Scale ranging from 0= "never" to 4= "almost always" - Recall period: Past month - There is no child report version of the Peds QL Stem Cell Transplant Module validated for children ages 5-7. | - Reliability: Both the generic PedsQL and the SCT-specific scales have shown high internal consistency, with Cronbach alpha >0.70 - Construct validity: Summary scores of the generic PedsQL and the PedsQL Stem Cell Transplant module are strongly correlated (r = 0.89 in children and r = 0.81 in parent proxy assessments) - Discriminates children with and without cGVHD: Scores of pediatric patients with cGvHD were significantly lower than the scores of patients without cGvHD (79.2 vs. 90.4; p ≤ 0.01); the scores of parent reports of patients with cGvHD were significantly lower than the scores of parent reports of patients without cGvHD (77.6 vs. 88.9; p ≤ 0.01) |
| Lee cGVHD Symptom Scale (LSS) (Lee et al., 2002; Mitchell et al., 2010; Merkel et al., 2016; Lee et al., 2018) | - Measure of cGVHD-associated symptom bother for use with adults age 16 or older - Assesses the degree to which respondents were bothered by each of 30 cGVHD symptoms - Responses choices: 5-point Likert scale; (“no symptoms or not bothered at all, slightly bothered, moderately bothered, bothered quite a bit, or extremely bothered”) - Recall period: Past month - Produces seven subscales (energy, skin, nutrition, muscles/joints, breathing, psychosocial, eye/mouth) - Subscales scores are linearly transformed (0-100) and summed to create a summary score of cGVHD-specific symptom bother | - Reliability: As expected with a summative symptom inventory, since symptoms fluctuate from day to day, test-retest reliability was modest (r=0.64). Internal consistency reliability was high (Cronbach’s alpha r=0.90). Individual symptom subscales with the highest internal consistency such as ocular symptoms (r=0.93) and mouth symptoms (r=0.87) demonstrated stronger test-retest reliability. - Construct validity: Weak correlations between selected subscale scores on the cGVHD symptom scale and unrelated domains of the Medical Outcomes Study Short Form-36 (SF-36) and Functional Assessment of Cancer-BMT (FACT-BMT). Energy subscale correlated most strongly with physical health domains on the SF-36 and FACT-BMT, whereas the psychological subscale had the strongest correlations with scores for emotional function on the FACT-BMT and the mental health subscales of the SF-36 |
